# Coloring Book: A new method for testing language comprehension

**DOI:** 10.3758/s13428-018-1114-8

**Published:** 2018-09-04

**Authors:** Manuela Pinto, Shalom Zuckerman

**Affiliations:** Utrecht University, Utrecht, The Netherlands

**Keywords:** Language comprehension, Grammatical development, Vocabulary assessment, Ecological validity, Task-related effects, Processing

## Abstract

We present a new method for investigating children’s language comprehension and argue that it has the potential to mitigate known task-related biases and expose children’s grammatical and lexical knowledge in a more natural and ecologically valid manner. The new method consists of filling in a digital coloring page, according to sentence stimuli (e.g., The green monkey is being scratched by the blue monkey; The rabbit is red.). Through the playful act of coloring in the page, children reveal their interpretations of grammatical constructions and lexical items. We argue that this method gives more accurate results than existing methods, in which children are asked to choose among several pictures representing a number of possible interpretations. We present two experimental studies: one with Dutch-speaking children, tested on four types of grammatical constructions, and a second study with children learning Dutch as a second language, tested on their vocabulary knowledge. In both studies, the new method was compared with one of the most widely used methods: the picture selection task. In the first study where children’s performance is said to be underestimated, the new method reveals better performance whereas in the second study where children’s performance is assumed to be overestimated, the new method reveals lower performance. The results suggest therefore that the new task indeed decreases external task-related effects and offers a more reliable measurement of children’s linguistic knowledge.

## Introduction

Current offline methods for assessing language comprehension offer subjects several possible interpretations, presented as pictures, and ask them to select one. Such methods are popular in many areas of language assessment—for example, in experimental studies that seek to evaluate the grammatical or lexical knowledge of normally developing children of a certain age group (e.g., Brown, 1971; Crain & Thornton, 2000; Gleitman, Gleitman, Landau, Wanner, & Newmeyer, 1988; see Schmitt & Miller, 2010, for an overview); in diagnostic tests designed to assess the comprehension of individual children, either normally developing or impaired (e.g., Bishop, 1979; Friedmann & Novogrodsky, 2011; van der Lely, 1996); and in evaluative tests of the proficiency level and progress of second-language learners (e.g., Slabakova, 2008; White, 2003). Following Adani (2011), Crain and Thornton (2000), de Villiers and de Villiers (2010), Schmitt and Miller (2010), and many others, we argue that such methods can lead to inaccurate assessment of the subject’s knowledge. Specifically, we argue that the explicit presence of alternative interpretations is unnatural and can affect the subject’s initial intuition.

We present a new offline method for the study of language comprehension, at both the grammatical and lexical levels, and discuss its advantages in comparison with existing methods. The new method consists of a coloring task in which subjects are asked to fill in the items on a digital page, resembling a regular coloring book, according to a specific sentence with coloring instructions. Subjects’ performance reveals their interpretation of the sentence without explicitly exposing them to different possible interpretations, which are nevertheless present in the coloring page. This means that subjects are not distracted by interpretations they consider irrelevant and “see” only the interpretation(s) they allow. The coloring task is presented with a digital touchscreen application called Coloring Book, a playful tool that adds a natural sense and ease to the task and increases the task’s ecological validity.

The main purpose of this article is to supply theoretical arguments for the applicability and possible advantages of the new method. The results from two experimental studies with Dutch-speaking children are presented as empirical support. In Experiment 1, we tested four grammatical constructions, for which claims of underestimation due to task effects, processing load, and other nonlinguistic factors have been made in the literature. The results show that the Coloring Book method yields significantly more adult-like^1^ performance than a picture selection task. In Experiment 2 we tested the vocabulary knowledge of immigrant children who had only recently come in contact with the Dutch language. For such children, claims of overestimation have been made. The results show that the Coloring Book method yields significantly lower performance than the picture selection task. Crucially, we show that these lower results are both more valid and more reliable in estimating children’s real vocabulary knowledge level.

This article is structured as follows: In the next section, we take a closer look at the challenges and goals of language comprehension research and at the type of data we need in order to reach these goals. In “Coloring Book: Presenting a new offline task” section, the new Coloring Book method is presented and compared with other existing methods. In “Experiment 1: Comparing the CB task and the PST in testing the comprehension of grammatical constructions” section, we present an experimental study on grammatical knowledge that compares the new method with an existing method, the picture selection task. In “Assessing vocabulary using Coloring Book” section, we discuss the advantages of using the Coloring Book method for vocabulary assessment, and in “Experiment 2: Comparing the Coloring Book and the Picture Selection Task in testing vocabulary” section, we present an experimental study testing vocabulary. Finally, “Conclusion” section sets out a critical discussion of the implications and the range of possibilities offered by the new method.

## An inherent obstacle when investigating language comprehension

When studying language comprehension, be it of young children, second language learners, or individuals with language or other cognitive impairments, the investigator faces an immediate challenge. Whereas in language production the object of investigation—the spoken or written sentence—is “out there” and ready to be analyzed, the interpretation that a subject assigns to a given text or utterance is not directly recoverable. In these cases, a mediating task cannot be avoided—that is, a task that indirectly measures the subject’s interpretation. Since the object of investigation is language itself, we cannot simply ask subjects to describe what they understand of a specific sentence, since this would bring them to a metalevel of linguistic analysis, which may not be the specific purpose of a study on language comprehension (e.g., Schwartz, 1993; VanPatten & Rothman, 2015; VanPatten & Williams, 2014; White, 1991). Furthermore, simply asking for the subjects’ interpretation involves production, rather than only comprehension, which again can lead to misestimation of the subjects’ knowledge. Indeed, in the present study we focused on measuring the comprehension of grammatical structures. A claim frequently made is that particular populations (e.g., young children) do not display competence with certain grammatical constructions, and their poor performance on specific comprehension tasks is exhibited as evidence of their lack of competence. Consider, for instance, a passive construction such as “The zebra is being scratched by the monkey.” The competence of correctly interpreting such structures may be underdeveloped in young children (Fox & Grodzinsky, 1998; Maratsos, Fox, Becker, & Chalkley, 1985; Snyder & Hyams, 2015), and even less developed in children with language impairments (van der Lely, 1996). Similarly, the interpretation of the pronoun in “The monkey scratches him” is governed by structural rules (Chomsky, 1981) that appear to be problematic for young children in certain languages—the well-known *delay of principle B effect* (DPBE; Chien & Wexler, 1990). The claim is made that children do not have adult-like comprehension for other grammatical structures, including object relatives (Brown, 1971; Friedmann, Belletti, & Rizzi, 2009), adjunct and complement control constructions (McDaniel & Cairns, 1991), and many more. The aim in assessing the comprehension of such constructions is to reveal the mental image that subjects form when hearing or reading these sentences, while diminishing various biases and task effects as much as possible.

Language comprehension can be assessed by means of online or offline methods. Online methods, which are assumed to examine subjects’ implicit preferences, measure the reaction to a stimulus in an indirect way, by relying on observation of a specific unconscious performance (reaction time, head turning, eye movement, brain activity, etc.). In addition, online methods are dependent on laborious and time-consuming analyses, and they require highly specialized equipment, involve a high level of “noise,” and do not allow assessment on an individual level. Therefore, the picture these methods give of the subject’s competence may not be exhaustive.

### Existing offline methods: Ecological validity versus controllability

Offline methods for studying language comprehension can be divided into three main categories: the picture selection task (PST; Gerken & Shady, 1996), the truth-value judgment task (TVJT; Crain & McKee, 1985; Gordon, 1996), and the act-out task (Ferreiro, Othenin-Girard, Chipman, & Sinclair, 1976). All three methods have been extensively used and have provided fruitful data. However, all three have also been criticized for several shortcomings (de Villiers & de Villiers, 2010; Hirsh-Pasek & Golinkoff, 1991; Katsos & Bishop, 2011; McDaniel, McKee, & Cairns, 1998; Schmitt & Miller, 2010). In many cases, researchers who use them sense that the data these tasks provide do not always reflect the subject’s full range of knowledge of the investigated structures. Furthermore, these methods do not yield consistent results across studies.

The act-out task (AOT) is the most ecologically valid of the three methods, and it is considered by many to be the most direct way to ask subjects about the meaning of a specific sentence without using language. This task consists of performing—literally, acting out—the meaning of the stimulus by using a set of provided props. This method requires no intermediate steps in order to measure other activities, and it allows subjects to project their own intuitive interpretation of the stimulus. Furthermore, it reflects a possible daily-life activity, which is especially relevant for children. The price of this ecological validity, however, is lack of controllability. The open-ended nature of this task creates a serious limitation to the analysis of its results; subjects are free to act out any scenario, limited only by the props provided to them, their own level of spatial awareness, and their creativity and acting talent. This liberty can result in broad variation in the manners that subjects choose to perform the task. Personality traits such as shyness, lack of confidence, hyperactivity, creativity, ambitiousness, and so forth, can all affect the outcome. In addition, there is a level of subjectivity in the interpretation that the experimenter assigns to the subjects’ performances. These issues likely account for the fact that AOT is the least commonly used of the three methods.

The PST and the TVJT increase controllability, since they offer the subject a closed and small set of possible responses to choose from. In a PST, subjects must match sentences with pictures. First they hear (or read) a stimulus, and then they are presented with two or three pictures from which they need to select the one that matches the stimulus. In a TVJT, the stimulus sentence is accompanied by a single picture, and subjects are asked to judge whether the picture correctly represents the sentence (match condition) or not (mismatch condition). Both methods represent useful devices to tap into language comprehension and language competence. Both can also be easily adapted to serve different populations. However, as is often the case, when controllability increases, ecological validity decreases. These tasks do not reflect natural linguistic activities—neither the comparing nor the evaluating of interpretations is an activity that children (or adults) do under normal circumstances. These activities are, rather, typically test-related, and their artificial settings make the outcomes of these tests vulnerable to nonlinguistic factors such as yes/no bias, lack of self-confidence, and the like.

### A problem: Explicit presence of alternatives

In this section, we wish to develop this point and concentrate on one less-discussed drawback of such methods as the PST and the TVJT for testing language comprehension, which we will call the *explicit presence of alternatives* (EPA) problem. Under normal circumstances, when subjects hear a sentence, they immediately assign an interpretation to it. Although some evidence exists that, in the process of interpretation, several alternatives are considered before one is selected, there is no dispute that this process of comparison and selection is automatic and that subjects are unaware of it (Grodzinsky & Reinhart, 1993; Just & Carpenter, 1992; MacDonald, Pearlmutter, & Seidenberg, 1994). The PST brings this process to an explicit level in the most obvious way—that is, by offering the available alternatives, so that subjects are forced to make a conscious choice, even when they would not ordinarily be aware of these alternatives. Additionally, the TVJT requires subjects to evaluate a given interpretation by explicitly comparing it to their own intuitive interpretation. The mismatch condition, in particular—in which subjects are presented with a less conventional or ungrammatical interpretation and asked to compare the two representations in an explicit manner—is crucial in a TVJT. As such, this kind of evaluation task is, in fact, a comparing and selecting task, in which subjects explicitly compare the suggested interpretation with their intuitive interpretation. One critical difference between the PST and the TVJT, as is discussed in Crain and Thornton (2000), has to do with the type of knowledge being tested. Although both methods test the intuitions and preferences of the subjects and show which grammatical structures their grammar allows, only the TVJT, via the option of rejecting a suggested interpretation, can test which structures are *not* allowed by their current grammar. This advantage of the TVJT is sometimes criticized as testing children’s tolerance for pragmatic violations rather than their linguistic competence (Katsos & Bishop, 2011). In this article, we concentrate on a method’s ability to reveal subjects’ (first) intuitions and preferences. (At the end of “Coloring Book: Presenting a new offline task” section, we will briefly return to this point in light of the newly proposed task.)

An additional negative effect of the EPA is that it increases the processing load. The cognitive operation of comparing, evaluating, and selecting alternatives has a processing cost, which makes it more vulnerable to interference from factors of a different nature than the natural operation of intuitively assigning an interpretation to a sentence. Engelhardt, Ferreira, and Patsenko (2010) showed that exposing subjects to a visual interpretation that conflicts with their own intuitive interpretation leads to higher processing load. Especially with vulnerable populations, such as children and language-impaired subjects or with second-language speakers, such an increase in processing load might lead to poorer results and, therefore, to underestimation of the subjects’ real capabilities. Thus, explicitly presenting alternatives affects the interpretation process, making it less natural. Adani (2011) discussed this point in a study of the comprehension of relative clauses by children. As in Hickok and Avrutin (1996) and Friedmann, Belletti, and Rizzi (2009), Adani suggested including all options in a single event, in order to decrease the explicit selection process. For example, a picture shows a horse chasing a lion that, in turn, is chasing a horse, and the child is asked, “Show me the horse that the lion is chasing.” Adani demonstrated that this single-event presentation does, in fact, improve children’s performance with regard to object relatives, indicating that the explicit selection process might indeed lead to underestimation of children’s knowledge. Nevertheless, all such testing items feature a similar three-character chain event, and the way in which the alternatives are presented in these single-event pictures is still partially explicit, so that they are limited in their use. In another study, Adani and Fritzsche (2015) compared results from an offline PST (explicit) and online eye-movement measurements (implicit) and showed that the PST yields lower performance rates, possibly because of the explicitness of the task. In a recent study, Frizelle, O’Neill, and Bishop (2017) also examined preschool children’s performance on relative clauses using two different tasks, picture selection and sentence repetition. Their findings also show poorer performance on the PST. They too concluded that the presentation of multiple pictures causes confusion and might lead to underestimation of children’s knowledge.

Another attempt to address and solve the EPA problem can be found in Huang, Spelke, and Snedeker (2013). In their attempt to shed light on children’s interpretation of number words, the authors proposed the “covered box task,” which calls for a number of interpretations (pictures) to be presented to children, while an additional picture is covered. Children are asked to select the correct picture out of the visible ones, but if they cannot find the correct picture among the visible ones, they can ask to look at the covered picture. This is an attempt to solve the EPA problem by offering alternatives in a semi-explicit way, but it has obvious disadvantages, in that the experimenter must decide which interpretations should be made visible and which ones should remain hidden. Furthermore, children’s performance might be driven by curiosity rather than linguistic intuitions.

Thus, the heart of the EPA problem is this: Although a test sentence may be ambiguous (carrying several possible interpretations), the pictures used are always unambiguous, and therefore several pictures must be presented to match the different possibilities. To overcome the EPA problem while maintaining controllability, we need to offer a sort of “ambiguous picture”—one in which several interpretations are available, but in an implicit way. In the next section, we propose such a task.

## Coloring Book: Presenting a new offline task

**Fig. 1:**
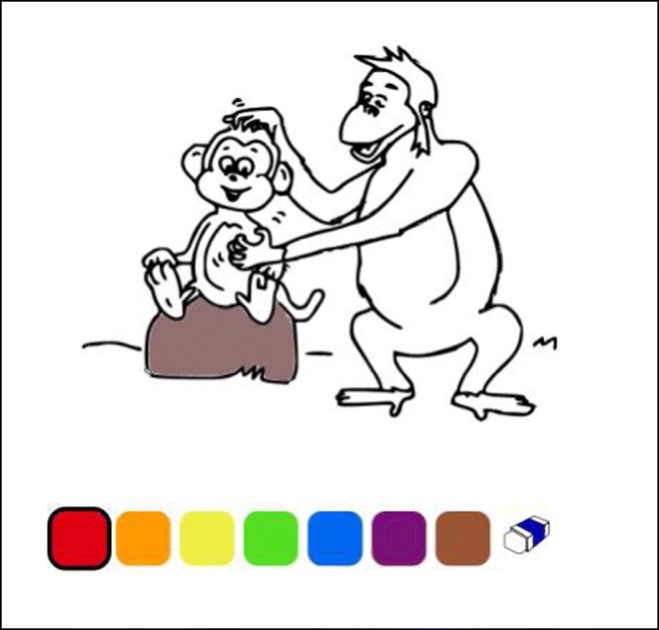
A coloring test item for passive constructions. The red monkey is being scratched by the blue monkey

In this article, we present a new task that aims to solve the EPA problem and increase ecological validity without losing controllability. This is a coloring task in which subjects are asked to color characters on a coloring page according to a set of instructions. See the example in Fig. 1.

The coloring page presents the activity as a single event in which no selection or comparison is necessary. Nevertheless, several possible alternatives are “hidden” in the page, and the subject is free to project her own intuitive interpretation through the act of coloring. In Fig. 1, the adult-like interpretation (*blue scratches red*), the non-adult-like interpretation (*red scratches blue*), and a “filler”—that is, an irrelevant interpretation (*red scratches yellow*, or any other irrelevant colors)—are all possible choices for the subject. In this way, the requirement of offering the subject all plausible alternatives (Crain & Thornton, 2000; Hamburger & Crain, 1982) is met. Crucially, the subject is not explicitly exposed to the multiple interpretations, and thus the possibility of interference is decreased. The coloring task remains ecologically valid, since it requires a simple, natural, and known activity and does not require specialized instruments or laboratories. Indeed, this method might best be described as a combination of an act-out task in a controlled picture selection environment.

The coloring task is administered in Coloring Book (CB), a Web application developed by the Digital Humanities Lab at Utrecht University. Using a touch-screen tablet, children do not actually need to color the characters. Rather they touch the color they desire in the on-screen palette, then simply touch the area they wish that color to fill. This platform presents the experiment in a playful and natural way that allows for a decrease in tension and “test feel,” thus diminishing potential biases and helping children reveal their natural and most intuitive preferences and choices. Additionally, once children have colored an item, they see the result of their choice (the colored items on the page) and can confirm it (by going to the next item) or correct it (by erasing their choice and coloring a different item or using a different color). This is another crucial advantage of the CB method over the PST and the TVJT, neither of which presents children with a visual consequence of their choice and lets them evaluate and confirm or change their choice. In a PST or a TVJT, children simply point, or say yes or no, and then move on to the next section.

Finally, the CB method allows for assessing language comprehension without using language (i.e., the answer to the stimulus is not provided by means of words or sentences). This property makes the CB method adequate for testing populations in which language production has not yet fully developed (e.g., toddlers and preschoolers) and in cases of delayed or impaired language production (e.g., child second language [L2] in the *silent period*, specific language impairment patients, or aphasics, among others). However, perhaps less obviously, this new method also could help making language assessment in bilinguals and L2 learners more objective and reliable. As is well known, the control of cross-linguistic interference is one of the major difficulties in testing bilingual populations: Even if they are in a monolingual mode, subjects may involuntarily activate the other language and show a distorted picture of their real linguistic competence (Grosjean, 1998). Furthermore, particularly in adult L2 learners, a linguistic response may activate the explicit competence of the L2 grammar (the L2 *normative* grammar), which may not be the goal of the study. The coloring task can also be easily adapted to adult populations by modifying the style of the drawings and by presenting the test as a *color memory* test.

Summarizing, the advantages of the Coloring Book method include the following:

- **Implicitness** Subjects are not explicitly aware of the various possible interpretations, although these are present and equally accessible.
- **Naturalness** Coloring is a natural activity, which children do in daily life.
- **Intuitiveness** Subjects assign the first interpretation to the sentence that comes to their mind, as in natural circumstances.
- **Lower processing cost** The cognitive load using CB is assumed to be lower as it does not involve explicitly comparing alternatives.
- **Activeness** Subjects are required to perform an active operation (coloring) unlike the picture-pointing or yes/no responses required in other methods, increasing the validity of the coloring method because subjects cannot respond without understanding the task or paying attention to it.
- **Self-evaluation** Subjects get a visual result of their choice and can confirm or change it.
- **Well-formed input** Subjects are exposed only to grammatical and valid interpretations. (For example, this is not always the case in grammaticality judgment tasks and in the mismatch condition in truth value judgment tasks).
- **Hidden purpose** Because coloring is the primary activity, it is easier to hide the linguistic purpose of the task from subjects.
- **Controllability** Subjects are restricted to a very limited possible set of actions
- **Playfulness** The coloring task is experienced as a game rather than a test, helping to decrease anxiety and improve performance in subjects with low self-esteem and fear of failure.

### A brief note on the CB vis-à-vis the TVJT

As we mentioned above, the TVJT has one essential advantage over all other methods: It can test not only whether children accept (and prefer) the correct interpretation, but also whether they reject the incorrect one. The Coloring Book method does not currently offer this possibility. However, we are working on a supplement to the method: the color correction task, in which a number of coloring pages appear with several precolored items. Children hear a sentence and are asked to keep (confirm) or change (reject) the items that are already colored. We plan to test and present this addition to CB in the near future.

We claim that the advantages outlined above help decrease common biases and produce more accurate measurements of subjects’ comprehension of grammatical constructions. In the next section, we put this claim to an empirical test with young children and show that for specific grammatical structures, the coloring task yields significantly more adult-like results than does the PST.

## Experiment 1: Comparing the CB task and the PST in testing the comprehension of grammatical constructions

Comparing methods, particularly methods intended to assess language comprehension, is not an easy task, since we do not have an objective measurement by which we can evaluate the methods. The hypothesis that led to this experimental study capitalizes on the assumption that by controlling a number of nonlinguistic factors—for example, processing load, experimental biases, naturalness, and so forth—we should be able to obtain a more accurate measure of the subjects’ linguistic competence. In this sense, a cleaner method will not necessarily provide higher scores, but rather scores that better reflect the subjects’ linguistic knowledge. Nevertheless, in order to be able to make a prediction, one can concentrate on structures for which the suspicion of underestimation due to nonlinguistic factors has been proposed. For such structures, one can hypothesize that a method that mitigates biases and external effects will yield higher scores.

We selected four such structures and tested them using both the PST and the CB method. Sixty Dutch preschool and young school-aged children participated in the study. Each child was tested with the same items, using both the existing PST method and the new CB method. The tested structures were: verbal aspect inflection, passive constructions, object pronouns and anaphors, and subject pronouns. For all structures, the assumption (based on previous results from the literature) was that preschool children already command the relevant rules, but for various reasons do not always perform in an adult-like manner. In such cases, the expectation is that methodological improvements would reveal more adult-like performance. Specific details and a review of the relevant literature for each of the tested grammatical structures are given in “Materials” section.

The primary purposes of the experiment were to

1. Confirm the validity of the CB method, predicting a significant correlation between the PST and CB, and
2. Show that children’s general performance in the tested structures is significantly more adult-like with the CB than with the PST, predicting an effect of “method” in a general linear mixed-effect model.

### Subjects

Sixty Dutch-speaking children (ages: 3;11–8;7, where, e.g., 8;7 indicates 8 years 7 months; mean age: 6;6) participated in the experiment. The children were grouped into four age groups, according to the (pre)school class they attended. All children were members of the same public primary school in a small town in the Netherlands. Informed consent for the experiment was obtained from the school authorities and from the children’s parents.

### Materials

**Fig. 2:**
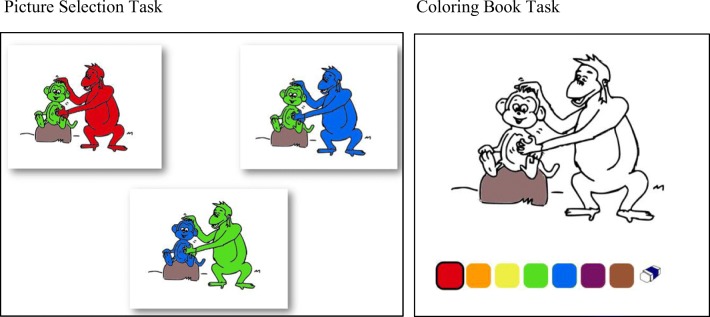
Example of a test item for the PST and CB methods. De groene aap wordt gekrabd (door de rode aap). The green monkey is scratched (by the red monkey)

Each child completed two test sessions, one with the PST and one with the CB. Both sessions included the same 24 prerecorded sentences, accompanied by the same drawings; however, in the CB test, the drawing was presented as a single coloring page via the iPad application, whereas in the PST test, the same drawing was presented in three versions, each version precolored in a different way. (See Fig. 2.)

The 24 items in the test represented the four grammatical structures: verbal aspect, passive, pronouns/anaphors, and subject pronouns. There were three conditions for each of these grammatical structures, reported in detail in Table 1.

**Table 1.** The tested structures

Grammatical Topic	Conditions	Examples
Aspect	Present	***De rode clown doucht.*** *The red clown showers.*
	Past	***De rode clown heeft gedoucht.*** *The red clown showered.*
	Future	***De rode clown gaat douchen.*** *The red clown is going to shower.*
Passives	Active	***De groene aap krabt de rode aap.*** *The green monkey scratches the red monkey.*
	Short passive	***De groene aap wordt gekrabd.*** *The green monkey is scratched.*
	Long passive	***De groene aap wordt gekrabd door de rode aap.*** *The green monkey is scratched by the red monkey.*
Binding	Reflexive	***Een groene aap zit op een steen en een rode aap krabt zichzelf.*** *A green monkey is sitting on a stone and a red monkey scratches himself.*
	Pronoun (salient antecedent)	***Een groene aap zit op een steen en een rode aap krabt hem.*** *A green monkey is sitting on a stone and a red monkey scratches him.*
	Pronoun (nonsalient antecedent)	***Een groene aap zit op een steen er is ook een rode aap. De rode aap krabt hem.*** *A green monkey is sitting on a stone and there is also a red monkey. The red monkey scratches him.*
Subjects	While (embedded)	***Een kleine kip loopt achter een grote kip, terwijl ze/die een bruine worm opeet.*** *A small chicken is walking behind a big chicken while she is eating a brown worm.*
	And (coordination)	***Een kleine kip loopt achter een grote kip en ze/die eet een bruine worm op.*** *A small chicken is walking behind a big chicken and she is eating a brown worm.*
	Period (intersentential)	***Een kleine kip loopt achter een grote kip. Ze/Die eet een bruine worm op.*** *A small chicken is walking behind a big chicken. She is eating a brown worm.*

For each of the grammatical structures examined, there were six items. Each child received two items per condition in a Latin square design, resulting in three versions (1, 2, and 3). In the *subject pronoun* condition, the items were further divided into two additional categories—sentences with the pronoun “ze” and sentences with the pronoun “die” (see Table 1)—resulting therefore in six different versions of the test (1a, 1b, 2a, 2b, 3a, and 3b). Each version was seen by ten children, and every child received the same version with the CB or the PST.

A short description of each of these structures follows.

- **Aspect:***The red clown showers/showered/is going to shower.*

The main question here is whether Dutch preschool children have mastered three of the most common verbal aspects used in Dutch: simple present (“doucht”/*showers*), past perfect (“heeft gedouchd”/*has showered*), and future inchoative (“gaat douchen”/*is going to shower*). Previous studies had indicated that Dutch-speaking children have mastered these constructions by the age of 4, but nevertheless they do not always reach adult-like performance levels, possibly due to task-related considerations. Preschoolers have been specifically reported to experience difficulty with comprehension of the future “gaat” structures: When presented with three pictures corresponding to the three time aspects, they sometimes incorrectly select the picture reflecting the ongoing event as matching the future “gaat” structure (Jordens, 1990; Wijnen & Verrips, 1998; Zuckerman, 2013). These structures are also known to be problematic for language-impaired children (de Jong, Blom, Orgassa, van de Craats & Verhagen, 2013; Zwitserlood, van Weerdenburg, Verhoeven, & Wijnen, 2015). Claims that non-adult-like performance on comprehension of verbal inflection is due to task effects has also been demonstrated for German (Brandt-Kobele & Höhle, 2010). The verbs that were used with this structure were *shower*, *jump*, *eat*, *play*, *paint*, and *walk*.

- **Passives:***The green monkey is scratched (by the blue monkey).*

The main question here is whether preschool children will show knowledge of two forms of the passive voice, the long (untruncated) passive, which includes a *by* phrase, and the short (truncated) passive, which lacks a *by* phrase. Although there is consensus that preschool children perform well in the comprehension of these structures, the results are often lower than adult-like level. Some studies (Horgan, 1978; Verrips, 1996) have claimed that the long passive is more problematic then the short one. Active sentences are used in this category as control items. Specifically, there is consensus that children have more difficulty with passives of psychological verbs (e.g., *see*, *hear*, *miss*, *remember*) than with action verbs (e.g., *scratch*, *lift*, etc.; Fox & Grodzinsky, 1998; Maratsos et al., 1985; Snyder & Hyams, 2015), although the range and degree of this difficulty remains under dispute. For this reason, we included two psychological verbs among the test items. Here, too, claims have been made that children’s comprehension is underestimated due to task-related effects (e.g., Messenger, Branigan, McLean, & Sorace, 2012). The verbs used with this structure were *scratch*, *make wet*, *lift*, and *bite* (action verbs), as well as *see* and *miss* (psychological verbs).

- **Binding (Principle B):***The green monkey*_*i*_*scratches him*_i__*_/himself_*i*_.

The main question here is whether preschool children will allow a local reference with a pronoun, which is prohibited by principle B of binding theory. Following Chien and Wexler (1990), Avrutin and Wexler (1992), McKee (1992), and many others, Philip and Coopmans (1996) and Baauw (2013) have shown that Dutch children also perform in a non-adult-like manner and allow such local reference up to 5 or 6 years of age. Recently, claims have been made that this Delay of Principle B-effect (DPBE) is caused or enhanced by methodological factors (Conroy, Takahashi, Lidz, & Phillips, 2009; Elbourne, 2005) or processing-related factors (Baauw, Zuckerman, Ruigendijk, & Avrutin, 2011; Hendriks, Van Rijn, & Valkenier, 2007). This structure is therefore a good test case to see whether children would perform better with a new method. An additional claim regarding DPBE is that antecedent saliency considerations may also play a role (Grimshaw & Rosen, 1990; Grodzinsky & Reinhart, 1993). For this reason, two pronoun conditions were included, as shown in Table 1. Regarding reflexives, there is consensus that preschool children comprehend them, although children often do not score 100% with them (see, e.g., Chien & Wexler, 1990). The verbs that were used with this structure were *scratch*, *lick*, *bite*, *make wet*, *put on makeup*, and *take a picture* (all of which are single-word transitive verbs in Dutch).

- **Subject Pronouns:***A small chicken*_*i*_* is walking behind a big chicken*_*j*_*, she*_*i/j*_* is eating a brown worm.*

This structure is somewhat different from the previous three, since it involves a discourse preference rather than a grammatical constraint. The main question here, therefore, is whether children will show preferences similar to those of adult native speakers in assigning a reference to the ambiguous subject pronoun. Two pronouns, opposing in the reference associated with them, were used. The pronoun “ze” (*she*) is ambiguous in these constructions, but most often, in the absence of other factors, is associated with the subject of the main clause (the topic). The pronoun “die” in these structures is said to refer exclusively to the object of the main clause (the focus). Whether this operation is a grammatical constraint or a (very strong) discourse preference is under dispute (see Van Kampen, 2010). A preliminary pilot study with these sentences showed that adult native Dutch speakers had a 100% preference for the subject as antecedent when the pronoun was “ze,” and a 100% preference for the object as antecedent when the pronoun was “die.” The three conditions in this category represent three different structural relations between the clause containing the antecedent and the following clause containing the pronoun: an embedded clause (*while*), a coordinate clause (*and*), or intersentential (separated by a period). These structural differences have been claimed to affect the choice of the antecedent (Carminati, 2005). Also for these structures that are governed by pragmatic constraints, it has been claimed that biases, task effects, and processing load may affect the procedure used for reference selection, particularly in children and L2 learners (e.g., Koster, Hoeks, & Hendriks, 2011; Sorace, 2011, 2012). The verbs that were used with this structure are *wave*, *eat*, *juggle*, *dance*, *hold*, and *eat up.* Table 1 gives an overview of the tested structures.

Given the fact that children’s performance with these four linguistic constructions is said to be affected by nonlinguistic/experimental factors, we predicted that the CB method, mitigating these interfering factors, would improve children’s scores.

### Procedure

Each child was tested in a quiet room on school premises. (In the Netherlands, preschool classes and elementary school classes are located in the same building.) In the first week, each child was tested with one of the two methods, and in the following week with the other method. Half of the children started with the PST and half with the CB. In both sessions, the same test version was used with every child, and the same prerecorded sentences were played in the same order using a laptop. In the PST session, each sentence was accompanied by three precolored pictures (see Appendix A for examples of the test items). Children had to point to the picture that best fit the sentence, and the choice was noted by the experimenter. In the CB session, each sentence was accompanied by a single coloring page. Children had to select a color from the color palette by touching it and then apply the color to the object of their choice by touching it as well. In both methods, children could ask to repeat the prerecorded sentence if they wished to, without limit.

### Results

Children received a score of 1 for a correct answer, for a total score from 0 to 24 for each of the two methods. (For the subject pronoun items, a score of 1 was given when the answer was similar to the preferred adult interpretation: i.e., when “ze” referring to the subject antecedent and “die” to the object antecedent.)

The results were analyzed with IBM SPSS Statistics, version 24. On the basis of an outlier analysis, two children in the youngest age group were excluded from the experiment, since their results (combined for both methods) were significantly lower than the group’s average. Furthermore, the experimenters confirmed that these two children seemed not to pay full attention or understand the tasks. The results are therefore based on the scores of 58 children (3;11–8;7, mean age 6;7).

**Fig. 3:**
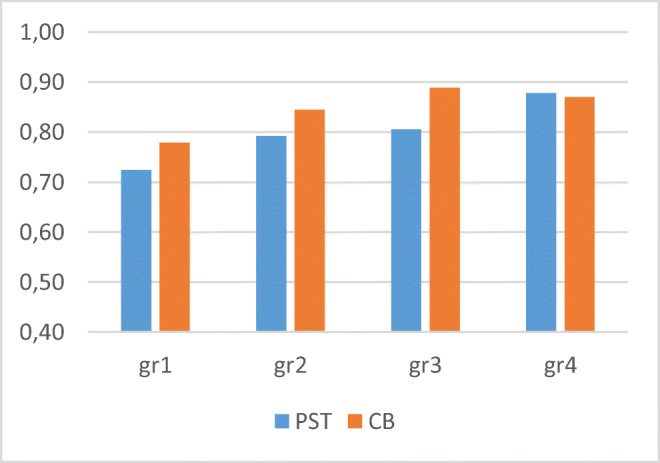
Mean results in Experiment 1 for each method, by age group

The average score for the test was 19/24 (80%) adult-like responses with the PST and 20/24 (85%) with the CB (mean advantage = 5%, *SD* = 9%). The individual data (Appendix B) reveal an advantage of up to 25% with CB. A Pearson correlation analysis between the scores of the two methods revealeed a significant correlation, *r* = .625, *p* < .01. Of the 58 children, 29 (50%) scored higher with CB (mean CB advantage = 12.8%), whereas only 18 (31%) scored higher with PST (mean PST advantage = 5.6%); 11 of the children (19%) performed equally well with both methods. Table 2 and Fig. 3 present the mean results by age group.

**Table 2.** Mean results in Experiment 1 for each method, by age group

Group	*N*	Age (mean)	PST	CB
1	13	3;11–5;6 (4;10)	.72	.78
2	15	5;7–6;6 (5;11)	.79	.84
3	15	6;7–8;0 (7;0)	.81	.89
4	15	7;8–8;7 (8;1)	.88	.87

A generalized linear mixed-effect model (GLMM) with method as a fixed effect and group, name, and sentence as random effects reveals a significant effect of method, confirming that scores with the CB method were higher (*F* = 11.845, *df* = 1, *p* < .01). Furthermore, the GLMM showed no significant effect of order of testing (i.e., whether CB or PST was conducted first; *F* = 0.308, *df* = 1, *p* > .05) and no effect of version (*F* = 1.780, *df* = 5, *p* > .05).

**Fig. 4:**
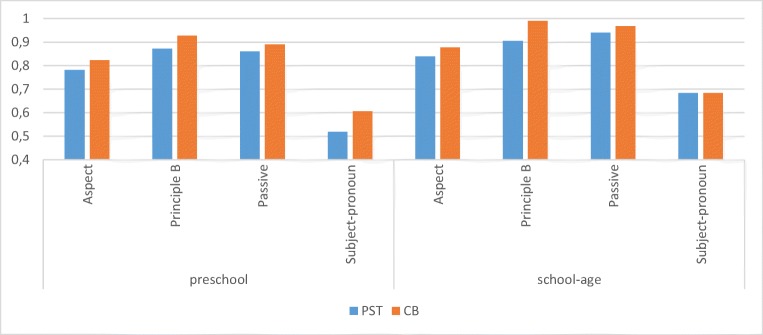
Mean correct (adult-like) answers for each topic, by age group

As can be seen in Table 2 and Fig. 3, the advantage of CB is visible in Age Groups 1–3, but not in Age Group 4 (ages 7;8–8;7). We believe that this is the result of a ceiling effect; the scores of these children are already very high (mean score 87%), and thus they gain no advantage through the method. Additionally, a significant negative correlation can be seen between the mean combined score for both tests and the CB advantage (Pearson – .285, *p* < .05; see Appendix B). This supports the claim that children who perform at a high level in general have less of an advantage with the CB, whereas children who score relatively lower in general benefit more from the new method. The GLMM further showed a significant interaction of method and grammatical topic (condition nested in grammatical topic: *F* = 8.532, *df* = 11, *p* < .01). See Fig. 4 for the results for each grammatical structure. The amount of data per topic/condition does not allow here for a proper quantitative analysis of each condition separately. Nevertheless, for illustrative purposes, in the following subsection we will try to speculate on the contribution of CB to each condition, by comparing the CB results to existing results from the literature.

**Fig. 5:**
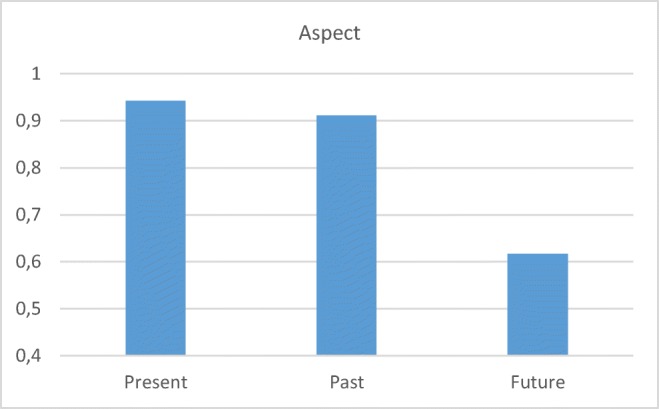
Preschool children’s mean correct responses with CB for the three aspect conditions

**Fig. 6:**
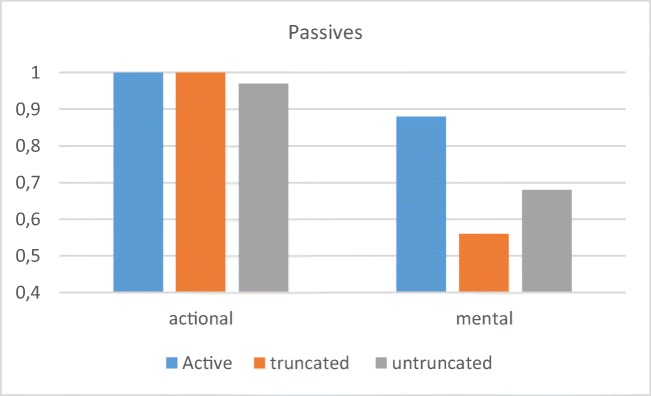
Preschool children’s mean correct responses with CB for the three passive conditions, divided into action and mental passives

**Fig. 7:**
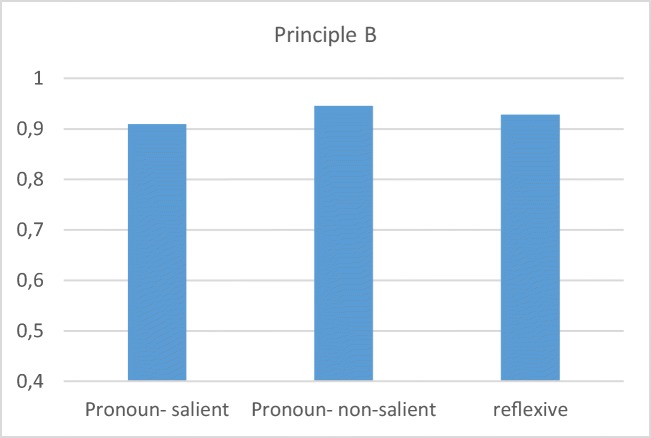
Preschool children’s mean correct responses with CB for the three principle B conditions

**Fig. 8:**
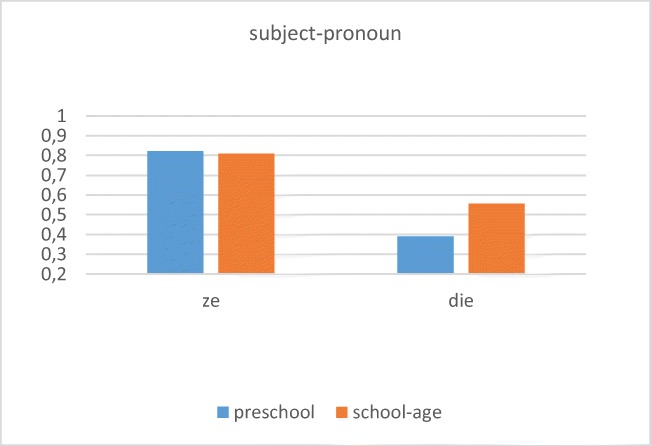
Preschool and school age children’s mean adult-like responses with CB for the two pronoun types

The CB results for each condition, presented in Figures 5, 6, 7, and 8, in several crucial cases reveal performance that seems to be different (more adult-like) than has previously been reported in studies conducted with other methods. Only the preschool children’s results (*n* = 28) are presented in most cases, since these represent the ages and data relevant for comparison with previous studies.

The results of the present, past, and future conditions are all higher (more adult-like) than was reported in, for instance, Zuckerman (2013), where children of comparable ages scored around 60% correct on present and past and 30% correct with “gaan” structures. The CB results further confirm the existing claims (Jordens, 1990; Zuckerman, 2013) that Dutch-speaking preschool children have trouble interpreting “gaan” structures and consider them to represent present tense.

The CB results show virtually perfect performance on actional passives from the youngest ages. Previous studies on actional passives have often shown good performance, but rarely with such clear numbers. For example, Hirsch and Wexler (2006) reported 81% correct responses with truncated passives, and 71% with untruncated, using a PST. Crucially, performance on mental passives was higher than has previously been reported (~ 40% in Hirsch & Wexler, 2006), suggesting that preschool children are able to interpret such structures when tested with a more sensitive method. Another important difference between the present CB results and results from previous studies is that with the CB, no difficulty was reported with “long” untruncated passives, which some have claimed (Horgan, 1978; Verrips, 1996) to be more difficult.

Here, too, the preliminary CB results here indicate better performance with pronoun (principle B) structures than has previously been reported. The previous difference between the interpretation of pronouns and reflexives in these constructions is not attested with the CB method, in which children perform above 90% correct in all conditions. These results accord with recent claims (Conroy et al., 2009) that experimental considerations, rather than a lack of knowledge, are responsible for the DPBE.

For the subject pronoun conditions, the present results with the CB method reveal a clear difference between Dutch children’s knowledge of the subject pronouns “ze” and “die.” The results indicate that preschool children do not yet recognize the function of “die” as pointing to the object antecedent. This knowledge increases with age, but even by ages 7 and 8, many children do not yet perform in adult-like fashion with these constructions.

### Discussion

The significant correlation between the CB and the PST results supports the validity of the new method as an assessment tool for language comprehension. Given that the tested grammatical constructions receive relatively high results using both methods, the advantage of using the CB method and its most significant effect may be in diminishing the underestimation caused by the biases and task effects associated with using the PST. An alternative explanation, namely that PST’s lower scores reflect children’s true performance level, would imply that the CB overestimates children’s linguistic competence. This alternative hypothesis is less plausible, for two reasons: First, as we argued in “Coloring Book: Presenting a new offline task” section, the CB is a straightforward and transparent method that does not interpose additional layers between the stimulus and the response. As such, it should not have any (positive or negative) effect on the test. Second, the four structures we examined were specifically selected for their vulnerability to task effects, providing scores that have been claimed to lead to underestimation of the real linguistic competence of the tested subjects. On the assumption that these claims are correct, we predicted that better scores in these structures tested with the CB should be ascribed to the “improvements” of this new method.

An additional support for the claim that CB corrects underestimation caused by the PST is given by the significant negative correlation between the combined test results of each child (which may be taken to represent the level of the child, independent of method) with their CB advantage. Such a correlation is to be expected, we claim, within a group with a relatively high level of knowledge. If lower performance is due to task effects, then children who score lower should gain more from a “cleaner” method. Under the alternative hypothesis, that children’s performance is inflated by CB, no such correlation would be expected.

We conclude, therefore, that the tested children, as a group, had already mastered grammatical knowledge regarding the tested structures. Failure to perform at an adult-like level was due, rather, to external considerations, such as biases, experimental effects, or processing load—not to a lack of grammatical knowledge. Although each of the four constructions should be examined in more detail (i.e., with more items per condition) to be able to give a conclusive statement about children’s linguistic competence, our results seem to indicate that for the tested children, the required grammar is in place.

## Assessing vocabulary using Coloring Book

In the previous sections we have discussed and tested the advantages of the CB method for assessing grammatical knowledge. In this section we claim that the CB method also has clear advantages for testing lexical knowledge.

In his well-known “gavagai” example, Quine (1970) observes a fundamental indeterminacy of reference between words and their meaning. When a person points to a rabbit and says the word *gavagai*, we cannot be certain of the word’s exact meaning. In addition to obvious options, such as *running*, *look!*, *quick*, or other words associated with rabbits, Quine offers *rabbithood* and *undetached rabbit part* as possible meanings of *gavagai*. These possibilities are indistinguishable from the actual meaning—rabbit—even after an infinite number of observations of persons saying *gavagai* while pointing at different rabbits in different states. This basic problem of lexical meaning, learning, and translation can also be seen as a fundamental problem of lexical assessment.

The PST discussed above, in which subjects are asked to point to the correct picture, is by far the most commonly used method for assessing lexical knowledge in children (PPVT; Dunn & Dunn, 2007), the language-impaired (CELF; Semel, Wiig, Secord, & Langdon, 2006), aphasics (Boston Naming Test; Kaplan, Goodglass, & Weintraub, 2001), and, in many cases, bilinguals and L2 learners, as well. In most of these tests, subjects hear a word and are asked to point to the correct picture out of several options. As in Quine’s example, when children are tested for the word *rabbit* and they correctly point to the picture of the rabbit, we still cannot be sure that they are able to separate the rabbit from its parts or from other features or actions closely associated with rabbits. A critical requirement of the lexical PST is that the test items must be depicted in a standalone fashion (otherwise, it will not be clear what it is that subjects are pointing at). In many cases, however, this is impossible—think of such words as *fingernail*, which cannot be depicted without a finger; *ceiling*, without walls and other room elements; or *beak*, which would be hard to recognize without the bird. This shortcoming of the PST and the pointing method can lead to *overestimation* of children’s knowledge, unlike the cases discussed in the previous sections. Namely, the child may point to the correct item while actually not knowing the true meaning of the word.

**Fig. 9:**
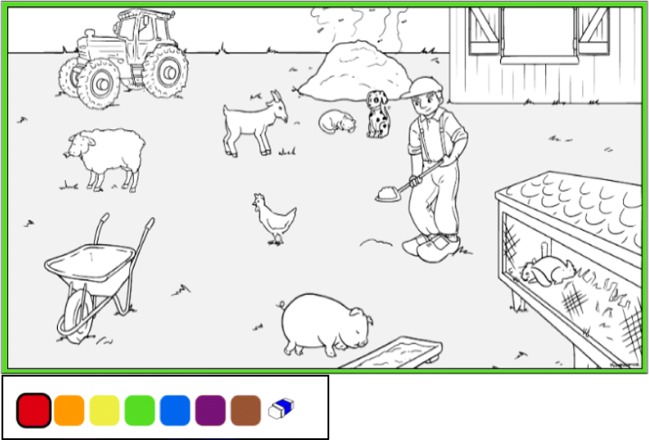
Testing vocabulary with CB

The coloring task offers an interesting solution to these problems. Subjects are presented with a coloring page that displays several items. The instruction is not given in the usual question or command form (e.g., *Point to the rabbit*, *Show me the rabbit*, or *Where is the rabbit?*) but as an invitation to color the items so that they match the statements provided (e.g., *The rabbit is blue*). The subject colors an item by choosing a color from the palette at the bottom of the screen and touching that item on the touchscreen. The item now has color, and subjects can view and confirm their choice. The colorable areas in the picture are predefined and may include all items, as well as background and parts of items. Figure 9 shows a coloring page for testing vocabulary.

This procedure allows us to test words such as *fingernail* (the subject can color only the finger, only the nail, or both) and *ceiling*. Furthermore, given the word *rabbit*, children can color the whole rabbit or only parts of the rabbit, and more importantly, they can confirm their choice. This is not possible when using the standard PST.

An additional advantage of using the coloring task for testing lexical knowledge is that it allows us to depict test items in context. Evidence shows that the context in which words are learned and tested has a fundamental effect on the way we store, interpret, and remember them (Bransford & Johnson, 1972; Maguire, Hirsh-Pasek, & Golinkoff, 2006). In a standard PST test, the item must appear independent of context, which can result in underestimation of subjects’ lexical knowledge. Children might very well know, for example, what an *electricity plug* or a *knee* or a *whisk* is, but fail to demonstrate this knowledge when presented with a picture of a plug without a wall or a knee without a person or a whisk without a kitchen. One can argue how much context should be given in a vocabulary test, but only the CB task allows for context—and indeed, for adjusting the amount of it. Furthermore, since the coloring task presents relevant objects as single items, not entire pictures, more items can be included in a setting. For example, a PST setting will test a specific word, say *tractor*, by presenting three or four pictures to select from (e.g., the picture of a tractor, a truck, and one or two more items, not necessarily belonging to the context of a farm). Conversely, in the CB the setting can include many more items in a natural context—for instance, a more fully realized picture of a farm, with a tractor, a barn, a farmer, and many animals (Fig. 9). Thus, the CB setting offers many more competing items, significantly reducing the chance of getting a correct answer by guessing, from 25% with a PST (four options) to 8%–10% with the CB (10–12 options).

Finally, the CB setting allows for presentation of the tested word within a natural grammatical sentence (*The tractor is blue*), where tests based on the PST often use single, standalone words (*tractor*) or ungrammatical constructions (*Show me “tractor”*). The grammatical structure can function as a cue, assisting children in revealing their full knowledge (Pearson, Hiebert, & Kamil, 2007; Read, 2000). This advantage is even more clear when we consider testing verbs (*Show me “running”* in a PST test), adjectives (*Show me “narrow”*), and prepositions (*Show me “under”*). The coloring method allows us to test all these items in their natural grammatical context, as parts of well-formed sentences (e.g., *The green monkey is running*, *The narrow path is yellow*, *The apple under the table is green*).

These advantages of the CB in testing lexical knowledge can lead in many cases to poorer performance than in the PST. Especially, the high likelihood of guessing correctly with the PST (25% or even higher) and the blunt nature of the pointing method (pointing to the correct picture but actually meaning only a part of it) might lead to overestimation of children’s lexical competence, especially in the case of children with a low vocabulary level. In the next section, we put the CB method to an empirical test in such circumstances.

## Experiment 2: Comparing the Coloring Book and the Picture Selection Task in testing vocabulary

The purpose of Experiment 2 was twofold: first, to show that CB can also diminish task effects when testing lexical knowledge, and second, to show that CB does not always lead to higher performance in comparison to the PST, as in Experiment 1, but that it can also lead to poorer performance in cases in which PST causes overestimation. Cases of overestimation are more probable when testing populations with a low language proficiency level, because such subjects more often rely on external cues or (educated) guessing and take more advantage of task-related biases. The subjects examined in this study were the children of refugee and immigrant families who had only recently arrived in the Netherlands and were exposed to the Dutch language. These children were in a special program in which they were placed in a special *language school* for a maximum of 2 years. During this period, their language level was assessed in order to decide when they were ready to enter the regular education system. Apart from allowing us to test children with a low vocabulary level, this setting offered us two additional measurements of children’s progress, which are independent of both the PST and CB methods. First, we could keep track of children’s active vocabulary score (i.e., their production rather than comprehension), which was tested regularly by the school. Second, we could record the number of days the children had been in the language school, which can be seen as a unique measurement of the child’s exposure to Dutch. We used these two additional external variables to assess the validity of the two tested methods.

The predictions of this experimental study were therefore:

1.Confirming the validity of the new method, the CB scores should correlate significantly with the PST scores, as well as with the external variables—productive vocabulary and number of days in school.

2.Children should score lower on their vocabulary knowledge with the CB than with the PST (showing that the CB reduces overestimation due to guessing and to relying on external cues).

### Subjects

Fifty-four children, ages 4;2–6:10 (mean age 5;2), participated in the experiment. All children were newcomers to the Netherlands and attended a special program for immigrant and refugee children. The contact with the teachers was in Dutch. For each subject, we registered their age as well as the number of days they had attended the school—the range of this variable was from 47 to 543 days in school, with an average of 199 days.

### Materials

The language school evaluated the children’s progress several times during the school year. To measure vocabulary knowledge, children were usually tested with a PST designed by Logo 3000, a commercial company (http://www.logo3000.nl). This company provides schools and other educational institutions with an online database of 3,000 basic Dutch words, supplemented by drawings and photos that depict each word. The school uses this material to practice and test children’s vocabulary. Every few months, children’s receptive and productive vocabulary was tested by using 40 selected words (20 words for perception and 20 different words for production).

To compare the PST method (used by Logo 3000) with the CB method to assess receptive vocabulary, we designed coloring pages that included the same 20 words chosen by the school for the receptive test.

### Procedure

Each child completed three tests. The PST and the production test were administered by a teacher of the school (as a part of the periodic routine), whereas the CB test was done by a researcher. Twenty-five of the children were first given the PST test and a few days later the CB test, and 29 experienced the tests in the other order. The production test was conducted immediately after the PST receptive test (as a part of the school’s routine). In all three tests, a correct answer received a score of 1 and an incorrect answer a 0, leading to a total score from 0 to 20 for each of the three tests.

### Results

Children’s scores with the PST ranged from 30% to 100% correct answers (mean 73%), whereas their scores with the CB were poorer, ranging from 0% to 95% (mean 51%). For the production test, the scores ranged from 0% to 100%, with a mean of 50% of items that were named correctly. The individual results for all variables are presented in Appendix C.

Children’s PST and CB scores were highly correlated: *r* = .82, *p* < .01.

A *t* test for matched samples revealed that children’s performance was significantly higher with the PST than with the CB: *t* = – 10.73, *df* = 53, *p* < .01.

A Pearson bivariate correlation analysis revealed significant correlations between the two methods (the PST and the CB) and the external variables—productive vocabulary and number of days in school. To measure the strength of the relation between each of the methods and the external variables, a partial correlation was computed, holding in turn each of the methods constant. The bivariate and partial correlations are presented in Table 3.

**Table 3.** Bivariate and partial correlations in Experiment 2

	Bivariate Correlation		Partial Correlation (Controlling for Other Test Score)	
	Production	Days in School	Production	Days in School
CB	*r* = .881, *p* < .01	*r* = .647, *p* < .01	*r* = .647, *p* < .01	*r* = .437, *p* < .01
PST	*r* = .808, *p* < .01	*r* = .530, *p* < .01	*r* = .317, *p* < .05	*r* = – .01, *p* > .05

The internal reliability of the CB and PST tests was assessed by using Cronbach’s *α* (Cronbach, 1951). Both methods received a high Cronbach’s *α* value (CB = .893; PST = .822). A Fisher–Bonnet test for comparing two Cronbach’s *α*s (Kim & Feldt, 2008) revealed that the internal reliability of the CB test was significantly higher than that of the PST (*Z* = – 1.7886, *p* < .05).

### Discussion

The results of Experiment 2 confirmed the validity and reliability of the CB method for testing vocabulary. The significant correlations of children’s CB scores with the PST scores, and even more so with the external variables of productive vocabulary and number of days in school, support the use of the CB as a valid method for assessing children’s vocabulary. The high Cronbach’s *α* of the CB confirms the internal reliability of the method.

When comparing CB and the PST regarding the above-mentioned measurements, CB seems to have the upper hand in all cases: Its internal reliability is significantly higher than the PST’s, and its correlations with the external variables are also higher. Crucially, the partial-correlation data show that holding the PST scores constant does not affect the strength of the CB correlation with the external variables, but when CB scores are held constant, the PST correlation can disappear. This clearly implies that the CB scores are a better predictor of children’s vocabulary level than the PST.

Summarizing, in Experiment 1 we tested children with a high proficiency level in Dutch. In this case, the CB yielded better results than the PST. In Experiment 2 we instead tested children with low competence in Dutch. As predicted, in this case the scores with the CB method were poorer than those with the PST. These results confirm our hypothesis that in cases of low proficiency, the PST can lead to overestimation, mainly due to (educated) guessing and relying on task-related cues. Therefore, the results of Experiment 2 are complementary to those of Experiment 1 and support the claim that the CB method can give a more accurate picture of children’s proficiency than the PST does. In addition, the CB appears to be an appropriate method not only for assessing grammatical knowledge, but for vocabulary development as well.

## Conclusion

In this article we have discussed several structural shortcomings of existing methods that assess language comprehension by offering explicit alternative interpretations, and we presented the Coloring Book, a new coloring method that, we believe, overcomes many of these shortcomings. Through two experimental studies, we confirmed its validity and advantages as compared with the popular picture selection task, regarding the assessment of both grammatical and lexical knowledge.

It is important to reiterate that our claim is not that CB will lead to better performance, but to performance that more closely reflects the true level of knowledge of the tested subject. We claim that the PST, comparable to a multiple-choice question on a test, can lead to either underestimation or overestimation of test takers’ knowledge. Subjects with high levels of knowledge are more likely to be underestimated by the PST; if one knows the answer, multiple alternatives can be confusing. Subjects with low levels of knowledge, on the other hand, are more likely to be overestimated; if one does not know the answer, multiple alternatives can help in making an educated guess. By this analogy, the coloring method may be compared to a semi-open question in a test: It exposes subjects’ true level of knowledge and, thus, leads to better performance—but it does so only if subjects already possess the relevant knowledge, since it also precludes guessing. In Experiment 1 (see “Experiment 1: Comparing the CB task and the PST in testing the comprehension of grammatical constructions’ section), we tested young native speakers of Dutch on constructions it is agreed that children understand, but for which they fail, when tested, to reach adult-like results for external or methodological reasons. With the CB method, subjects showed better performance. In Experiment 2, we tested a population with a low proficiency level in Dutch, young immigrants and refugees attending a Language School, and we showed that the CB method neutralizes the possibility of educated guessing—leading to lower performance, which is nevertheless a more accurate reflection of the real lexical level of these subjects. Future studies should further support the validity of the CB method and explore situations in which it offers an advantage over existing methods.

Within the field of grammar assessment, many structures show non-adult-like performance, possibly due to task effects. Consider, for example, the comprehension of relative clauses, adjunct and complement control, quantifiers, bridging, negative polarity items, number modifiers, (scalar) implicatures, and so forth. In all these cases, research might gain more insight into subjects’ competence using the CB method.

Studies with special populations, such as children with specific language impairment, autism, dyslexia, Down syndrome, and other cognitive or linguistic disorders, can also benefit from use of the CB method. Asides from the advantages for typically developing children discussed in this article, children of such special populations are even more vulnerable to task effects. A user-friendly method such as the CB can open the way to a more accurate estimation of the comprehension level of these populations.

The facts that the CB can be presented as a memory task (in such a case, the sentence is presented first, and after a couple of seconds the coloring page appears) and that the task does not involve production open the possibility to use it with adult subjects as well. This is especially relevant for second language learners and bilingual subjects. Future studies should explore the applicability of the CB to testing and assessing these populations.

### Author note

We wish to thank Gali Freedman, Jason Rothman, Roumyana Slabakova, Yoad Winter and Sergey Avrutin for their inspiring and useful comments. We thank Emma Kooijman for the data collection and the teachers and children of the Taal school Utrecht for participating in the study. Many thanks to Digital Humanities, particularly to Julian Gonggrijp for creating the Coloring Book application and fulfilling all our digital wishes. Finally, we want to express our gratitude to three anonymous reviewers for their critical questions and insightful comments that greatly contributed to the quality of this article. This work is part of the research programme Alfa Meerwaarde, number 325-70-004, which is financed by the Netherlands Organisation for Scientific Research (NWO).

## Appendix A—Examples of test items with the picture selection task and Coloring Book

**Table:**

A1 – Aspect	De rode clown doucht. *The red clown showers*.
A2 – Passive	De groene olifant wordt natgemaakt (door de blauwe olifant). *The green elephant is sprayed (made-wet) (by a blue elephant)*.
A3 – Principle B	Een groene aap zit op een steen and er is ook een rode aap. De rode aap krabt hem. *A green monkey is sitting on a stone and there is also a red monkey. The red monkey scratches him*.
A4 – Subject pronouns	Een kleine kip loopt achter een grote kip, terwijl ze/die een bruine worm opeet. *A small chicken is walking behind a big chicken while she is eating a brown worm*.

## Appendix B Individual data in Experiment 1

**Table:**

Child *n*=58	Class	Age	PST 24 items	CB 24 items	Combined 48 items	CB advantage CB – PST
**1**	3	8:0	0,79	0,83	0,81	0,04
**2**	3	7:3	0,71	0,88	0,79	0,17
**3**	1	5:5	0,83	0,92	0,88	0,08
**4**	1	5:5	0,83	0,79	0,81	−0,04
**5**	4	8:3	0,92	1,00	0,96	0,08
**6**	4	7:9	0,67	0,79	0,73	0,13
**7**	1	5:3	0,79	0,83	0,81	0,04
**8**	1	3:11	0,50	0,63	0,56	0,13
**9**	4	8:2	0,83	0,79	0,81	−0,04
**10**	2	5:9	0,63	0,79	0,71	0,17
**11**	4	8:5	0,96	0,92	0,94	−0,04
**12**	2	6:6	0,79	0,79	0,79	0,00
**13**	2	6:3	0,71	0,79	0,75	0,08
**14**	3	6:11	0,63	0,88	0,75	0,25
**15**	4	7:8	0,92	0,88	0,90	−0,04
**16**	4	8:5	0,79	0,71	0,75	−0,08
**17**	1	4:4	0,67	0,75	0,71	0,08
**18**	1	4:1	0,54	0,75	0,65	0,21
**19**	4	8:2	0,79	0,92	0,85	0,13
**20**	1	4:7	0,79	0,79	0,79	0,00
**21**	4	7:8	0,96	0,92	0,94	−0,04
**22**	4	8:4	0,88	1,00	0,94	0,13
**23**	3	6:9	0,83	1,00	0,92	0,17
**24**	3	7:1	0,79	0,88	0,83	0,08
**25**	2	6:0	0,75	0,92	0,83	0,17
**26**	3	6:11	0,83	0,83	0,83	0,00
**27**	2	5:10	0,83	0,79	0,81	−0,04
**28**	2	5:9	0,67	0,92	0,79	0,25
**29**	4	8:3	0,96	0,96	0,96	0,00
**30**	1	5:6	0,71	0,79	0,75	0,08
**31**	2	5:11	0,83	0,79	0,81	−0,04
**32**	2	5:11	0,96	0,92	0,94	−0,04
**33**	2	5:9	0,92	0,92	0,92	0,00
**34**	3	7:1	0,75	0,88	0,81	0,13
**35**	2	6:3	0,71	0,83	0,77	0,13
**36**	4	8:7	0,96	0,88	0,92	−0,08
**37**	2	5:7	0,88	0,88	0,88	0,00
**38**	1	5:5	1,00	0,96	0,98	−0,04
**39**	4	8:2	0,88	0,75	0,81	−0,13
**40**	2	5:7	1,00	0,92	0,96	−0,08
**41**	1	4:1	0,54	0,67	0,60	0,13
**42**	1	5:2	0,75	0,83	0,79	0,08
**43**	2	6:2	0,71	0,79	0,75	0,08
**44**	2	6:1	0,83	0,83	0,83	0,00
**45**	2	6:0	0,67	0,79	0,73	0,13
**46**	4	7:8	0,88	0,79	0,83	−0,08
**47**	3	6:9	0,88	0,79	0,83	−0,08
**48**	3	7:3	0,79	0,96	0,88	0,17
**49**	3	6:11	0,71	0,67	0,69	−0,04
**50**	3	7:5	0,88	0,88	0,88	0,00
**51**	1	5:4	0,67	0,63	0,65	−0,04
**52**	4	8:2	0,92	0,92	0,92	0,00
**53**	3	6:6	0,92	1,00	0,96	0,08
**54**	1	4:10	0,79	0,79	0,79	0,00
**55**	3	7:1	0,88	1,00	0,94	0,13
**56**	3	6:7	0,79	0,96	0,88	0,17
**57**	3	6:9	0,92	0,92	0,92	0,00
**58**	4	8:0	0,88	0,83	0,85	−0,04
**ᅟ**						
**Outliers**	**Class**		**PST 24 items**	**CB 24 items**	**Combined 48 items**	
**59**	1		0,58	0,38	0,48	
**60**	1		0,42	0,5	0,46	

## Appendix C

**Table 4.** Individual results Experiment 2

	Age	Days in school	CB score 20 words	PST score 20 words	Production 20 words
**1**	4;2	68	0,1	0,55	0,06
**2**	4;6	186	0,6	0,95	0,83
**3**	4;10	305	0,4	0,85	0,33
**4**	4;10	278	0,6	1	0,56
**5**	4;11	292	0,75	1	0,89
**6**	4;4	82	0,45	0,85	0,39
**7**	4;7	229	0,75	0,95	0,78
**8**	4;6	61	0,6	0,95	0,56
**9**	4;9	102	0,6	0,95	0,61
**10**	4;11	334	0,9	0,95	0,61
**11**	5;9	250	0,8	0,95	1
**12**	5;1	61	0,2	0,5	0,33
**13**	4;6	82	0,55	0,75	0,44
**14**	5;0	327	0,8	0,9	0,83
**15**	4;8	250	0,65	0,8	0,56
**16**	4;11	299	0,7	0,95	0,83
**17**	4;11	61	0,15	0,6	0,33
**18**	4;8	82	0,9	0,6	0,72
**19**	4;6	102	0,35	0,6	0,06
**20**	5;3	249	0,7	0,85	0,83
**21**	4;3	68	0,1	0,4	0,06
**22**	4;9	249	0,6	1	0,89
**23**	4;4	68	0,1	0,35	0,11
**24**	5;9	47	0,15	0,5	0,17
**25**	5;6	61	0,1	0,6	0
**26**	6;10	145	0,7	0,75	0,56
**27**	5;8	82	0,8	1	0,61
**28**	6;6	292	0,6	0,8	0,39
**29**	5;4	145	0,5	0,8	0,56
**30**	5;5	327	0,55	0,75	0,61
**31**	5;10	439	0,85	0,9	1
**32**	5;9	180	0,4	0,5	0,17
**33**	6;7	543	0,65	0,75	1
**34**	6;4	299	0,6	0,8	0,78
**35**	5;10	68	0,25	0,35	0,22
**36**	5;9	166	0,3	0,6	0,28
**37**	5;10	157	0,4	0,45	0,44
**38**	5;6	173	0,6	0,8	0,56
**39**	5;10	145	0,05	0,5	0,11
**40**	6;0	341	0,75	0,8	0,72
**41**	4;11	87	0,6	0,9	0,61
**42**	4;7	173	0,25	0,45	0,06
**43**	4;10	305	0,9	0,95	1
**44**	4;4	82	0,25	0,5	0,33
**45**	4;10	299	0,95	0,85	0,67
**46**	4;11	348	0,6	0,85	0,83
**47**	4;4	82	0,4	0,6	0,17
**48**	4;2	81	0,1	0,55	0
**49**	4;7	54	0,25	0,6	0,17
**50**	5;7	362	0,85	1	0,83
**51**	4;10	145	0,25	0,5	0,06
**52**	6;7	407	0,85	1	0,83
**53**	5;6	75	0	0,3	0
**54**	6;2	432	0,6	0,6	0,56
**Mean**	**5;2**	**197**	**0,51**	**0,73**	**0,5**

## Footnotes

1 Throughout this article, we use the term *adult-like* instead of the term *correct* or *grammatical*, in order to make clear that we are not simply testing grammaticality but are more interested in disclosing the developmental process of language comprehension.
